# The Principles of Ophthalmic Surgery; Being an Introduction to a Knowledge of the Structure, Functions, and Diseases of the Eye, Embracing New Views of the Physiology of the Organs of Vision

**Published:** 1835-01-01

**Authors:** 


*The Principles of Ophthalmic Surgery; being an Introduction to a Knowledge of the Structure, Functions, and Diseases of the Eye, embracing New Views of the Physiology of the Organ of Vision.* By John Walker, Assistant Surgeon of the Manchester Eye Institution, &c.—London, 1834. Foolscap 8vo. pp. 195.

Mr. Walker is already known as the author of a small pamphlet, containing some original and ingenious views on the Physiology of the Iris; and the present publication will show that he has paid no less attention to the diseases than to the functions of the eye. This treatise, however, is so succinct, that it can only be looked upon as a compendium, excepting on a few topics, into which the author has entered at more length perhaps than is consistent with his general plan; but, as all his observations are those of a clever and thinking man, we tender him our thanks for his digressions, as nothing is a drier task than wading through, month after month, elementary treatises, scarcely varying in anything but the expressions in which they are couched.

The first point to which we shall direct our reader's attention is Mr. Walker's treatment of *purulent ophthalmia*. After describing very briefly the symptoms and causes of this disease, and mentioning the opposite plans of treatment that have been proposed by different writers, he thus proceeds:

"If the former line of treatment [*i. e.* the antiphlogistic,] be adopted, it is evidently applicable only to the first stage of the disease. In strong, plethoric persons, where the inflammatory action runs high, and is attended with febrile excitement, no possible harm can result from one moderate bleeding; but, to carry it to the point recommended by some persons, is exceedingly absurd, inasmuch as it will be highly injurious to the constitution of the sufferer, rendering it less able to struggle successfully through the after-stages of the complaint, or to repair the mischiefs which frequently result from it. My experience of this disease has led me to conclude that the subjects of it are not usually of that description in whom we should willingly order very copious venesection; indeed, the constitutional disturbance has never been at all marked; if anything, rather depressed than excited: in short, my impression of the disease is, that it is strictly local, and that it should be treated as such. If, therefore, the loss of blood be necessary, it appears to me that the local abstraction of it is to be preferred, and that it will be best effected by the application of a moderate number of leeches placed upon the eyelids, the drain from which will have a tendency to lessen the congestion of the vessels: that this practice will be often useful, I have no doubt. Counter-irritation by blisters, &c. may be of some benefit, but is not likely to influence the disease to any extent.

"The tartar-emetic seems admirably adapted to depress still further a state of constitution, which will soon be sufficiently depressed without it. Useful as this remedy may be in acute internal diseases, I should no more think of using it here than in any other ordinary local affection.

"That the antiphlogistic plan of treatment is inefficient, will appear from the want of success attending it. Dr. Vetch, who took fifty or sixty ounces of blood from patients affected with this disease, reports, that fifty cases, out of six hundred and thirty-six, lost the sight of both eyes, and that forty more were blinded in one eye, and this under every advantage, the patients being soldiers in a military hospital, and of course under strict surveillance. Most writers who advocate the antiphlogistic treatment, are compelled to admit that, in spite of its being employed most energetically, the disease will frequently terminate in destruction of the eye. I have myself seen many instances, where it has been duly followed up, in which it has completely failed to influence the progress of the disease, which has gone on to the destruction of both eyes. If therefore it has failed in the hands of individuals who have used it fairly and promptly, how much more likely is it to do so in those of practitioners in general, who in this disease seldom have recourse to more than half measures. The hundreds of people, old and young, who are constant victims of this destructive malady, speak, in a language that cannot be misunderstood, that our plans of treatment are anything but effectual in combating it; and, as the antiphlogistic (or something which goes under this name,) is the plan usually adopted, upon it only can we lay the blame, which attaches somewhere.

"If, on the other side of the question, we examine carefully the result of the stimulating treatment, we shall have ample reason to conclude, that, as the affection itself is a local one, so the remedies may be strictly local also. Dr. O'Halloran, who, as an army surgeon, had extensive opportunities of treating this disease, having become dissatisfied with his want of success from the antiphlogistic treatment, began the use of sulphate of copper applied to the inner surface of the lids, or dropped into them a ten-grain solution of nitrate of silver, which he generally repeated daily. He expresses his opinion that bleeding is unnecessary, and says that he has treated hundreds of cases with the stimulant treatment, and always with the greatest success.

"Mr. Guthrie has been very successful with an ointment in which the nitrate of silver is the prominent ingredient, and which is placed with a spatula or brush between the eyelids, so as to come in contact with the conjunctiva. Dr. Ridgway appears to have been the first to recommend the nitrate of silver in solution, in these cases.

"Feeling a similar dissatisfaction with the antiphlogistic plan, I resolved to have recourse to the stimulating treatment, in the very first case I had under my own exclusive care. I was of course aware that it had been used before; but, independently of that knowledge, this case immediately struck me as the one for this treatment. In a paper published in the Lancet, I gave a detailed account of the case, in which I had recourse to the nitrate of silver in substance, applying it to the inner surface of both eyelids once a day for the first week, and afterwards every second or third day, until the morbid state of the parts had disappeared. I have used it since then in many instances, in all ages, and in every stage of the complaint, with the most gratifying result. It can be applied with much greater facility than any liquid or unctuous substance. It is simply necessary to separate the lids, when eversion is sure to take place, and the pencil of the nitrate of silver is drawn lightly across the conjunctiva, which is but the work of an instant. The pain is not so severe as many persons may suppose, and soon subsides. The name, which is sometimes given to this substance, of *lunar caustic,* seems to have constituted it a complete bugbear. Its application is from this cause contemplated by some with a degree of horror, for which there is not the slightest occasion. It is in truth no caustic; for no destruction of surface, or at all events very slight, and consequently no cicatrization, ever results from its use.

"Little else need be done. Attention to cleanliness, and the frequent washing out of the matter from the surface of the eye, with a solution of alum or sulphate of copper, with attention to the general health, as may be required, constitute the essential points. It must be remarked, that, under the best kind of treatment, a certain period will elapse before the diseased action subsides. This will vary according to management, but in severe cases it will seldom be under six weeks." (P. 21.)

From this, our readers will perceive that our author is a warm opponent of the antiphlogistic treatment of these affections, and a no less sturdy champion of the stimulating plan; for, where other surgeons use a solution of nitrate of silver, he would recommend the substance. For our own parts, we are partly inclined to agree, and partly to differ, from his views: like him, we think the disease local, and, like him, we would apply our principal remedies locally; but we have seen so much benefit arise from the copious and frequent application of leeches to the eyelids, that we should be loth to resign their use, or even to employ them as sparingly as the author advises. With regard to the nitrate of silver, we believe it to be principally useful when the vessels have become relaxed by the continuance of the distension, and in such cases we would join our author in ascribing extraordinary powers to it; but we would mention a single case, that will tend to show that this disease is to some extent under the power of constitutional remedies. It fell to our lot to witness the practice of a surgeon, who had a very unusual method of treating affections of the eye. A patient came under his care, with a very severe attack of purulent ophthalmia, supposed to be produced by the contact of gonorrhœal matter, as he was at the time suffering from that disease. The eye was greatly swollen, the pain was extreme, and the conjunctiva so vascular, that it scarcely resembled the membrane usually known by that name; and there was considerable chemosis, especially about the edge of the cornea, which was injected with red blood, and showed signs of incipient sloughing. A few leeches were applied to the eye, and a drachm of the Vinum Colchici was exhibited three times a day. The next day the sloughing of the cornea was no longer equivocal, though the eye was somewhat less swollen. No more leeches were now applied, nor any other local treatment employed: the colchicum was continued; the slough of the cornea came away, but proved to be superficial; ulceration, however, went on, deeper and deeper, until the remaining layer was so thin as to begin to project, but the conjunctiva had become less vascular and swollen. Meanwhile the colchicum was acting violently, and the patient was greatly reduced by the vomiting and purging. Here then the disease stopped: this thin layer of the cornea never ulcerated through; cicatrization took place under the moderated use of the remedy, and, contrary to the expectation of every one who saw him, the patient perfectly recovered his sight, as the contraction of the cicatrix entirely removed it from the sphere of vision. We do not quote this case as a specimen of good surgery; but it evinces so satisfactorily the power of constitutional remedies over the disease, that it can scarcely be expedient to omit them, whatever may be our local treatment.

When ulceration or sloughing of the cornea has taken place in this affection, Mr. Walker recommends the application of nitrate of silver once or twice a day. We have never used it in substance, but can speak highly of the good effects of a strong solution, in these cases.

While speaking of the uses of the eye generally, our author makes some brief observations on the nature of light, which are not remarkable for the closeness of their reasoning. Thus, he says, "When we consider that it is not merely the medium by which other objects are seen, but that it is itself visible, and that it has also the property of not merely exciting, but irritating the eye, we are naturally enough led to believe that it is a real, material substance." (P. 42.) Let us make an application of a similar argument to the ear, thus: When we consider that sound has not only the power of exciting and irritating the ear, but that it may rupture the tympanum, and produce disorganization of structure, is it possible to believe that sound is not a real, material substance? We have no doubt that Mr. Walker would attribute these phenomena to the vibrations of the air; why not, then, the less marked effects of light to "certain undulations, communicated by luminous bodies to an ethereal fluid which fills all space." We do not pretend to settle the question of the nature of light, which has perplexed philosophers from the commencement of the world, and will most likely continue to do so till its end, but we cannot permit such loose talk to pass current as scientific argument; nor do we think that the author makes a better figure, while drawing an analogy between the opening and closing of flowers, and the natural tendency to keep the eyes open during the day, and closed during sleep: yet some of our brethren may feel grateful to him for so good an excuse for lying in bed late in the morning as the following passage will afford them; for, what use can there be in struggling against their vegetative nature?

"The tendency to close that the eyelids evince when in a state of darkness, and that to expand under the influence of light, may be considered, then, as bearing a striking analogy to the corresponding phenomena noticed in certain productions of the vegetable world.

"In the eye, a moderate degree of the stimulus of light seems to cause expansion; whilst an excess, as well as an absence, produces the opposite effect. It is well known that we are much more prone to sleep in dark winter mornings, and the reverse. It is the same in dull days: we feel a heaviness of the eyes and contiguous parts; and, to arouse ourselves into exertion, we employ friction over the face and eyebrows, as well as the eyelids, in order to stimulate the branches of the fifth pair, which are insufficiently excited by the diminished supply of light." (P. 44.)

The following observations, however, are of a very different stamp; and, for our own parts, we think that Mr. Walker's physiological views on the eye's sensibility to light are correct, and shall quote them, for the benefit of our readers.

"It has been the custom, however, to consider the retina as peculiarly the seat of sensibility to light; but many facts go to prove that this sensibility is not dependent upon the retina, but is derived from the fifth pair of nerves. In a case of disease of the fifth pair, where the taste, feeling, and voluntary motion, were all impaired, this sensibility to light was gone, although vision, the function of the retina, remained entire; and in paralysis of the retina, it is not unusual to observe this sensibility to light remaining. In M. Magendie's well-known experiments, after division of the fifth, he found the eye of the animal insensible to the stimulus of light. Hence he was led to imagine, that vision was destroyed by that injury, which was certainly a great mistake, arising from his not having made this distinction between sensibility to light and vision, two very different properties. Those experiments proved that the fifth has great influence over the eye; but to infer, as Magendie did, that it is therefore the nerve of vision, was carrying the induction farther than the premises warranted.

"Some individuals, supposing that this sensibility to light resided in the retina, have been unable to account for its possessing this quality, seeing that it has no connexion with the nerve of sensibility; hence Dr. Macartney was led to predict, that one day or other a branch of the fifth would be discovered penetrating to the retina. At present this prediction remains to be fulfilled.

"If the retina be sensible to the stimulus of light, and the contraction of the pupil be for the purpose of defending it from this stimulus, we might expect that, where no iris exists, a not unfrequent congenital deficiency, the intolerance of light would be extreme; whereas I should be inclined to say, from an instance I have myself observed, that it is imperfection of vision from the optical deficiency, rather than dread of light, which is experienced.

"The same may be said of the Albino. I recollect once asking a person of this description, whether the light was not very painful to her; it was a sunny day. Her reply was, 'No, it does not hurt me, but I cannot see so well." The terms, intolerance of light, and indistinctness of vision, are often used as synonymous, when speaking of these individuals. It is the optical deficiency which inconveniences them.

"In ordinary inflammation of the external parts of the eye, whether it arise from a particle of dust, or any other cause, and where the retina cannot possibly be affected, there is the greatest intolerance of light; whilst, in the most destructive internal inflammation, there is generally infinitely less dread of light, and sometimes none at all. It may be mentioned, as confirmatory of this view, that the pain in the eye, eyebrows, and head, which accompanies ophthalmia, is always diminished, by lessening the supply of light to the individual; and that the pain felt is always in those parts which are supplied by branches of the fifth.

"Persons who, from their occupation, expose their eyes to a strong glare of light, and others who inhabit high northern latitudes, such as the Tartars, where the light reflected from the snow is very injurious, producing what is termed snow-blindness, and which consists not in amaurosis, or affection of the retina, but in inflammation of the external tunics, are in the habit of defending the external parts of the eye by a frame, with a central aperture for the light to get to the retina. It is quite evident that the object of such individuals is to defend the sensitive, exterior portion of the organ, whilst as much light as ever must pass through the pupil to the retina. Indeed, if it be the retina that requires to be defended, the closure of the pupil must be the only effectual relief in these instances,—a process which would be incompatible with vision, and which never, under any circumstances, takes place.

"This view is further strengthened by the fact, as stated by the best authorities, that certain mammalia and reptiles, in whom the eye is in an imperfect, rudimentary state, and who have no optic nerve, and consequently no vision, have nevertheless a sensibility to light, which they receive from the ophthalmic branch of the fifth pair, the only nerve which enters the eye; such are the mole, shrew, mus capensis, proteus, &c. Many of the zoophytes also exhibit distinct indications of a sensibility to light, without the slightest trace of an organ of vision. In the annelida a near approach to an optical apparatus is found, but in so simple a form, that it is believed they possess merely a sensibility to light, and have no perception of objects.

"Thus it appears evident, from all these facts, that sensibility to light and vision are two distinct attributes, possessed by different portions of the organ of vision; that they are independent of, and may exist without, each other; and that the sensibility to light exists in the exterior of the organ, and is derived from the fifth pair, whilst vision is the function of the retina through the optic nerve." (P. 44.)

The above passage is a good specimen of the author's powers: as a piece of reasoning, the facts are clearly stated and well arranged, and, in our opinion, the conclusion is correctly deduced.

Mr. Walker is of opinion that near-sightedness depends upon an unnatural contraction of the pupil, and on an inability to accomplish its full dilatation. It is well known, indeed, that the pupil contracts when viewing a near object, and dilates when the eye is fixed on a more distant one; and any one who has read Dr. Roget's beautiful detail of his own case, as published in Mr. Travers's work, will probably be inclined to attribute the power of adjusting the organ of vision to different distances, partly, if not principally, to this contraction. But we cannot understand what the author gains by the mis-statements of the effect of glasses.

"In looking at remote objects the pupil always dilates, and the eyelids separate, to collect the rays from the distance. In near-sighted persons this is not sufficiently effected. The concave glass remedies this defect, by collecting the more distant rays, and, by converging them, allows their entrance into the pupil.

"Some individuals are able to see objects at considerable distances with distinctness, but find it difficult to discern near objects. To this state the name presbyopia is given. It is attributable to a want of proper contractility of the pupil, a process which always takes place in changing the eye from a distant to a near object. The opposite kind of glass, viz. the convex, is suited to this condition, for reading or looking at any near or small object, which has the property of representing these objects more distinctly, which it does by diverging the rays of light." (P. 66.)

We imagined that every schoolboy who had ever burnt a hole in his hat by means of a convex lens, was aware that, by its means, he collected the sun's rays into a focus, and that the reverse was accomplished by means of concave glasses.

We would willingly follow our author through all his arguments concerning the use of the iris, but the subject is extremely complicated; and a successful attempt to unravel it would require more than the limits of a review. Suffice it to say, that he is a believer in the muscularity of its structure, and that his opinion is founded upon the appearance of two sets of fibres which it frequently presents, and upon the fact of its being supplied with branches from a motor nerve. He affirms that the contraction of the pupil does not depend upon its sympathy with the retina, but with the third and fifth nerves. His reasons are as follows: 1st. As yet no connexion has been traced in the dead body between the ciliary branches and the retina. 2dly. In many cases of paralysis of the retina, and in perfectly opaque cataract, the iris remains active. 3dly. A dilated and motionless pupil sometimes occurs without loss of vision. 4thly. In sleep the pupil is contracted, though the retina is not excited. The principal objections which we have to urge against these statements are: 1st. The generality of cases of perfect amaurosis are attended by dilated and immoveable pupil, as it is this which gives the peculiar expression to the countenance of an amaurotic patient. 2d. When a pupil is dilated without disease of the retina, it is generally in consequence of disease of the iris itself. 3dly. Too little is known of the state of the nervous system during sleep, for any argument to be founded on the contraction of the pupil in that state. Lastly: It has been found by physiologists (and Mr. Walker does not pretend to deny it,) that the iris itself is insensible to light, as long as light is prevented from impinging on the retina.

Again, our author is of opinion, that the iris is connected by sympathy with the palpebræ, or, to use his own words, "that the sympathies and relations of the iris are with the palpebræ, and not with the retina;" and he adduces various circumstances as reasons for this hypothesis. 1st. They are supplied by the same nerves. 2dly. When the fifth nerve is divided, both iris and palpebræ are paralysed. 3dly. In certain cases of disease, in which there is a constant twitching and closing of the eyelids, there is also a rapid contraction and partial dilatation of the pupil; and, 4thly. That in the lower animals the iris is active in proportion to the perfection of development of the eyelids. Doubting, as we do, the first steps of Mr. Walker's argument, viz. that the sympathy of the iris is not with the retina, we cannot be expected to enter into any discussion upon these other statements in support of his hypothesis; but we may mention, *en passant*, that we have seen an instance of tremulous iris, in which there was no peculiar irritability of the palpebræ; nor do we think it surprising, that, in proportion as the eye is more perfectly developed, the action of its various parts, and of the iris amongst others, is likewise perfected; so that, we confess we are yet far from our author's conclusion, that the iris is but an internal eyelid.

Having thus completed our notice of the more interesting portions of the work, we think it due to our readers, and the author, to present them, before closing the book, with a specimen of the manner in which the diseases are treated of: and we open, as hazard directs us, on *Retinitis*.

"*Inflammation of the Retina. Active Amaurosis.*—Increased vascular action of the retina may vary in extent and severity, from simple turgescence or congestion, to the most active inflammation, which may be followed by deposition, or change of its structure, opacity, and even ossification, as is seen in some of the other textures of the eye, but which is not so visible here. It frequently arises suddenly, proceeds rapidly, and may destroy vision in a very brief period.

"*Symptoms.* The most important of these, and the one which first and most forcibly strikes the attention of the patient, is impaired vision, which may exist in various degrees, from slight dimness and obscurity, to complete and total blindness. Frequently also pain is complained of, deep in the orbit, sudden and severe darting into the head and neighbouring parts with a sense of confusion and giddiness of the brain. This however is by no means a constant attendant, as in the worst cases there is sometimes a total absence of suffering, particularly after the disease has existed some little time. Flashes of fire, and objects sometimes black, like flies, (*muscæ volitantes*,) at others red, sparkling, and variegated, are seen floating before the eye, with confusion, and change of form in surrounding objects. On looking into the pupil, we often observe, instead of the natural shining or brilliant black, a dull lack-lustre appearance, sometimes approaching to a grey colour, with an inactive, sluggish, or motionless state of the iris, frequently accompanied by a change in the shape of the pupil, which is oval, or still more irregular. The pupil is not always dilated, particularly at the onset of the disease, or, in the case of one eye only being affected, the iris often sympathising with the free action of its fellow. The impaired function of the iris is to be attributed to the extension of the diseased action to its structure, or from the ciliary nerves participating in, or being paralysed by, the inflammation of the contiguous membrane, the choroid, which we can hardly conceive to escape when there is any active disease of the retina. In the early stage there is sometimes intolerance of light, from the excited state of the organ, and occasionally this is the case in confirmed amaurosis; but more frequently in the latter, light makes no impression upon the eye, the patient not being able to discriminate between light and darkness.

" *Causes.* Frequent instances occur of active amaurosis arising suddenly, without any perceptible cause. Often it may be traced to direct causes, such as flashes of lightning, exposure to blazing lights and large fires, excessive use of the organ, particularly on minute or brilliant objects; intoxication, and general free living. More indirectly, the cessation of menstruation, suppression of hemorrhage, or other discharges, &c.

" *Treatment.* In the commencement very active measures must be resorted to, and those of an antiphlogistic kind. We must bleed largely from the arm, *ad deliquium.* This must be repeated in a short time, if no improvement takes place. Cupping, leeches, brisk purgatives, and counter-irritation, must follow, in quick succession, if we desire to check the progress of the affection. A pretty rapid use of mercury, so as to affect the mouth in a short period, or until the symptoms improve, is an essential part of the treatment of this disease. Perhaps this is the most important remedy. It should be used promptly and energetically, so that ptyalism be produced with as little delay as possible, when a restoration of the functions of the retina generally succeeds. A state of rest, with light diet, is to be advised, and the organ protected from light and other irritants, such as cold air, &c.

" If the attack be slight and the symptoms partial, or the patient feeble and delicate, the principle to act upon is still the same, but must be more cautiously and moderately put into practice. Here a smaller bleeding, perhaps a few leeches, with purgatives, counter-irritants, and a mild course of mercury, must be adopted, with an attention to the general health, or other circumstances that may arise." (P. 94.)

From what we have said, our readers will perceive that this is no ordinary work. It is made still more useful by the addition of a vocabulary of ophthalmological synonyms, in several languages.

We think that the treatment of diseases of the eye is on the eve of receiving many and most important improvements; and we have no hesitation in predicting that Mr. Walker will be eminently successful in enlarging our knowledge of this most interesting branch of the art of healing.

